# Ascorbate metabolism and the developmental demand for tartaric and oxalic acids in ripening grape berries

**DOI:** 10.1186/1471-2229-9-145

**Published:** 2009-12-09

**Authors:** Vanessa J Melino, Kathleen L Soole, Christopher M Ford

**Affiliations:** 1 The University of Adelaide, School of Agriculture, Food and Wine, Private Mail Bag 1, Glen Osmond, SA, 5064, Australia; 2 Flinders University, School of Biological Sciences, PO Box 2100, Adelaide, SA, 5001, Australia; 3 Current address: Centre for Rhizobium Studies, Murdoch University, South Street, Murdoch, WA, 6150, Australia

## Abstract

**Background:**

Fresh fruits are well accepted as a good source of the dietary antioxidant ascorbic acid (Asc, Vitamin C). However, fruits such as grapes do not accumulate exceptionally high quantities of Asc. Grapes, unlike most other cultivated fruits do however use Asc as a precursor for the synthesis of both oxalic (OA) and tartaric acids (TA). TA is a commercially important product in the wine industry and due to its acidifying effect on crushed juice it can influence the organoleptic properties of the wine. Despite the interest in Asc accumulation in fruits, little is known about the mechanisms whereby Asc concentration is regulated. The purpose of this study was to gain insights into Asc metabolism in wine grapes (*Vitis vinifera *c.v. Shiraz.) and thus ascertain whether the developmental demand for TA and OA synthesis influences Asc accumulation in the berry.

**Results:**

We provide evidence for developmentally differentiated up-regulation of Asc biosynthetic pathways and subsequent fluctuations in Asc, TA and OA accumulation. Rapid accumulation of Asc and a low Asc to dehydroascorbate (DHA) ratio in young berries was co-ordinated with up-regulation of three of the primary Asc biosynthetic (Smirnoff-Wheeler) pathway genes. Immature berries synthesised Asc *in-situ *from the primary pathway precursors D-mannose and L-galactose. Immature berries also accumulated TA in early berry development in co-ordination with up-regulation of a TA biosynthetic gene. In contrast, ripe berries have up-regulated expression of the alternative Asc biosynthetic pathway gene D-galacturonic acid reductase with only residual expression of Smirnoff-Wheeler Asc biosynthetic pathway genes and of the TA biosynthetic gene. The ripening phase was further associated with up-regulation of Asc recycling genes, a secondary phase of increased accumulation of Asc and an increase in the Asc to DHA ratio.

**Conclusion:**

We demonstrate strong developmental regulation of Asc biosynthetic, recycling and catabolic genes in grape berries. Integration of the transcript, radiotracer and metabolite data demonstrates that Asc and TA metabolism are developmentally regulated in grapevines; resulting in low accumulated levels of the biosynthetic intermediate Asc, and high accumulated levels of the metabolic end-product TA.

## Background

Ascorbate (Asc) is the most abundant soluble antioxidant found in plant cells and is present at various concentrations in nearly all fresh food. Since humans have, through evolution, lost the ability to synthesise their own ascorbate, it must be obtained from their diet [reviewed in [1]]. Asc, along with flavonoids, polyphenolics and lipophilic antioxidants, is often used as an indicator of the nutritional value of foodstuff [2]. Asc has been the focus of much attention due to the versatility of its cellular functions and its impact on plant growth and development, as reviewed by Smirnoff [3], De Gara [4] and Noctor [5].

**Figure 1:**
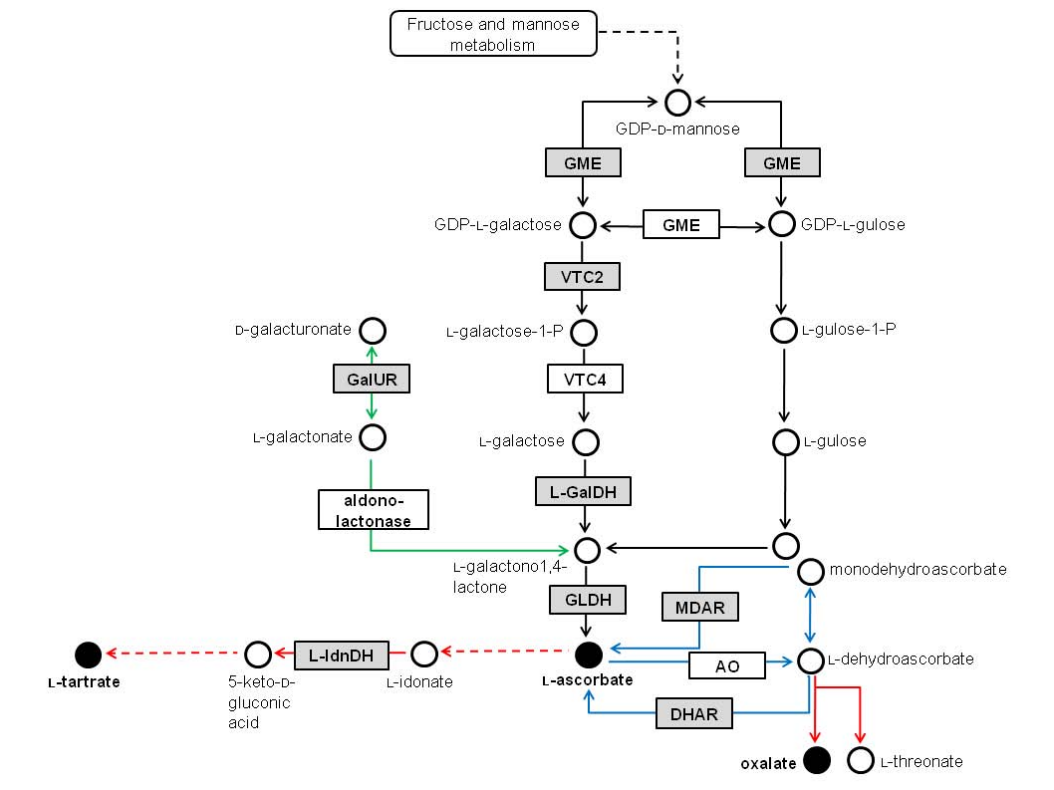
**The proposed pathways of L-ascorbate (Asc) metabolism in plants**. Single arrowed lines indicate one enzymatic step whilst dashed lines indicate multiple metabolic steps not shown in detail here. Black arrows represent steps in the primary Smirnoff-Wheeler Asc biosynthetic pathway, green arrows represent steps in the alternative 'carbon salvage' Asc biosynthetic pathway, blue arrows represent steps in Asc recycling and red arrows represent steps in Asc catabolism. Intermediates are represented by circles. Closed circles representing intermediates investigated in this study. The abbreviated names of enzymes catalysing individual steps are displayed in rectangular boxes. Shaded boxes highlight the genes encoding the enzymes investigated in this study. The Smirnoff-Wheeler primary Asc biosynthetic pathway enzymes include GDP-D-mannose-3,5-epimerase (GME), EC 5.1.3.18; GDP-L-galactose phosphorylase (VTC2), EC unassigned; L-galactose-1-phosphate phosphatase (VTC4), EC unassigned; L-galactose dehydrogenase (L-GalDH), EC unassigned; L-galactono-1,4-lactone dehydrogenase (GLDH), EC 1.3.2.3. The alternative Asc biosynthetic pathway enzymes include D-galacturonic acid reductase (GalUR), EC 1.1.1.203 and aldono-lactonase, EC 3.1.1-. Enzyme catalysed steps involved in recycling Asc include monodehydroascorbate reductase (MDAR), EC 1.6.5.4 and L-dehydroascorbate (DHAR), EC 1.8.5.1. C4/C5 cleavage of Asc in *Vitaceous *plants proceeds via the intermediates 2-keto-L-gulonic acid, L-idonic acid, 5-keto-D-gluconic acid, L-threo-tetruronate and L-tartrate. The only characterised enzyme of this pathway is L-idonate dehydrogenase (L-IdnDH), EC 1.1.1.264. C2/C3 cleavage of Asc or L-dehydroascorbate generates oxalate and L-threonate: this pathway may occur enzymatically or non-enzymatically.

Asc metabolism is also evident in the cytosol and in non-photosynthetic organelles including the mitochondria and peroxisomes. The enzyme L-galactono-1,4-lactone dehydrogenase, which is capable of synthesising Asc from L-galactono-1,4-lactone, is in fact bound to the inner mitochondrial membrane, in association with Complex I [6,7]. This enzyme is part of the Smirnoff-Wheeler Asc biosynthetic pathway, which is now widely accepted as the major pathway contributing to Asc accumulation in plants (Figure 1).

Wheeler et al. [8] demonstrated that D-mannose and L-galactose were effective precursors of Asc, interconverted by the activity of GDP-D-mannose-3,5-epimerase, an enzyme which has since been characterized in *Arabidopsis thaliana *[9]. Wheeler et al. [8] further isolated L-galactose dehydrogenase from cell free extracts of Arabidopsis leaves and pea embryogenic axes, which is capable of oxidising L-galactose to the final Asc precursor L-galactono-1,4-lactone.

Additional steps in the pathway were resolved using a different methodology from those just described; this was achieved by screening for ozone sensitive [10] and ascorbate deficient mutants [11] in *Arabidopsis thaliana*. *VTC1 *and *VTC4 *mutants were thus demonstrated to encode GDP-mannose pyrophosphorylase [12] and L-galactose-1-phosphate phosphatase [13,14], respectively. The *VTC2 *gene was more recently identified by two independent groups and described as a GDP-L-galactose/GDP-D-glucose phosphorylase [15] and a GDP-L-galactose:hexose 1-phosphate guanylyltransferase (EC 2.7.7.12) [16].

For many years, evidence has demonstrated the existence of an alternative Asc biosynthetic pathway (Figure 1) whereby D-galacturonic acid is converted to Asc by an inversion of the carbon chain [17-19]. Interest in this alternative pathway was revived by the cloning and characterisation of D-galacturonic acid reductase from strawberry fruit [20]. In this pathway, pectin derived D-galacturonic acid is reduced to L-galactonic acid. This intermediate is readily converted to the Smirnoff-Wheeler Asc biosynthetic pathway intermediate L-galactono-1,4-lactone [19], which is in both pathways converted to Asc by the activity of L-galactono-1,4-lactone dehydrogenase [21,22]. Another pathway for the synthesis of Asc has been demonstrated to occur from D-glucuronic acid, which is produced by the activity of *myo*-inositol oxygenase (MIOX) [23,24], but a recent report using *Arabidopsis *over-expressing *Miox *demonstrates that this pathway plays an insignificant role in Asc accumulation [25].

Intracellular Asc concentration varies between species and between tissues of the same species. For example, ascorbate concentration tends to be high in meristematic tissue such as in germinating seedlings [26,27] and in root apex cells [28]. The Asc content in fruit is also dependent on the tissue and the species [reviewed in [29,30]].

The biosynthesis of Asc is not the only factor regulating its cellular Asc concentration, Asc is also influenced by external stimuli such as nutrition [reviewed in [31]], light [32,33], temperature [34,35] and ambient ozone concentrations [36]. These stresses promote the formation of reactive oxygen species (ROS), which are removed by the plant's antioxidant system. The antioxidant system includes catalase, superoxide dismutase, peroxidases and enzymes involved in the ascorbate-glutathione cycle. This cycle includes ascorbate peroxidase (APX), monodehydroascorbate reductase (MDAR), dehydroascorbate reductase (DHAR), glutathione reductase (GR) and the antioxidants Asc and glutathione (GSH) [reviewed in [37,38]]. MDAR and DHAR specifically catalyse oxido-reductase reactions, which alter the balance of Asc to DHA (Asc recycling), Figure 1. The protective functions provided by ascorbate and related antioxidant enzymes against photo-oxidative stress in chloroplasts are reviewed in Noctor and Foyer [39] and in Foyer [40].

Investigating Asc accumulation in sink tissues such as fruit is further complicated by growing evidence that Asc translocation occurs to meet the demand for Asc in rapidly growing non-photosynthetic tissue. Franceschi and Tarlyn [41] demonstrated long-distance translocation of Asc from leaves to root tips, shoots and floral organs in the model plants *A. thaliana *and *Medicago sativa*. Further support for Asc translocation via the phloem from leaves to fruits or tubers has since been reported [32,42,43]. Ziegler [44] originally reported the presence of ascorbate in the phloem, and Hancock et al [45] identified ascorbic acid conjugates in the phloem of zucchini (Cucurbita pepo L.), which may play a role in phloem loading. However, the relative contribution of import on Asc accumulation in heterotrophic tissue has only been quantified in blackcurrants [46], and species differences are likely to exist.

Asc is not a stable metabolic end-product nor is it limited to oxido-reductase reactions that alter the balance of Asc to DHA; it can be catabolised to oxalic acid, L-threonic acid and L-tartaric acid [reviewed in [47,48]], Figure 1. In geraneaceous plants, Wagner and Loewus [49] demonstrated that cleavage of Asc between carbon atoms 2 and 3 results in the formation of OA from carbon atoms 1 and 2, and L-threonic acid (which may be further oxidised to form TA) from carbon atoms 3 to 6. The conversion or turn-over of DHA to oxalate/L-threonate via the intermediate 4-O-oxalyl-L-threonate was more recently reported [50]. In *Vitaceous *species, cleavage of the Asc catabolic intermediate 5-keto-D-gluconic acid between carbon atoms 4 and 5 leads to TA formation, with the two-carbon fragment of atoms 5 and 6 putatively recycled into central metabolic pathways [51-53]. Conversion of L-[1-^14^C]ascorbic acid to TA in young grapes has been demonstrated [54,55]. In a pathway distinct from TA biosynthesis, Asc is also cleaved in *Vitaceous *species between carbon atoms 2 and 3 leading to OA formation from carbon atoms 1 to 4. A more detailed review of the species differences between Asc catabolic pathways can be found in Loewus [56].

Unlike the oxido-reductase reactions that rely on Asc redox enzymes and non-enzymatic reactions to recycle Asc, catabolic reactions require continued Asc biosynthesis to replenish Asc lost to the synthesis of further compounds. In *Arabidopsis *leaves the loss or turnover of Asc is only about 2.5% of the pool per hour [57] whilst in embryonic axes of pea seedlings, the turn-over is about 13% per hour [58]. In flowers and early fruits, Asc turnover was low at 1.41% of the total Asc pool per hour and was increased with fruit maturity to 3% per hour [46,58]. The rate of Asc turnover in high oxalate or tartrate accumulators, such as in grapevines is yet to be established.

The purpose of this study was to investigate Asc accumulation and metabolism in grapevines, which unlike other higher plant species used in similar investigations, is an accumulator of both Asc degradation products, TA and OA. Genetic, biochemical and metabolite approaches were taken to study the various facets of Asc metabolism including Asc biosynthesis, Asc recycling and Asc turnover. In the present study, we demonstrate that both grapevine fruit and vegetative tissue can use D-mannose and L-galactose for the synthesis of Asc and for further metabolism to TA and OA. A quantitative analysis of the developmental fluctuations of Asc and its degradation products OA and TA in grape berries is presented here. Furthermore, we investigate developmental regulation of genes involved in Asc metabolism, and from this we highlight developmental differences between primary and alternative Asc biosynthetic pathways.

## Results

### Developmental accumulation of metabolites

Recently, a method for the simultaneous quantification of Asc, TA and OA was described and accumulation of each across four developmental stages was reported [59]. In this present study, the scope of the metabolite profile was extended to identify key physiological stages from pre-bud-break to harvest where correlative accumulation of the precursor and its catabolism products was evident: this was performed across two developmental seasons. The following berry analysis parameters enabled characterisation of specific physiological stages of development: fresh weight, sugar accumulation (total soluble solids) and malic acid accumulation.

**Figure 2:**
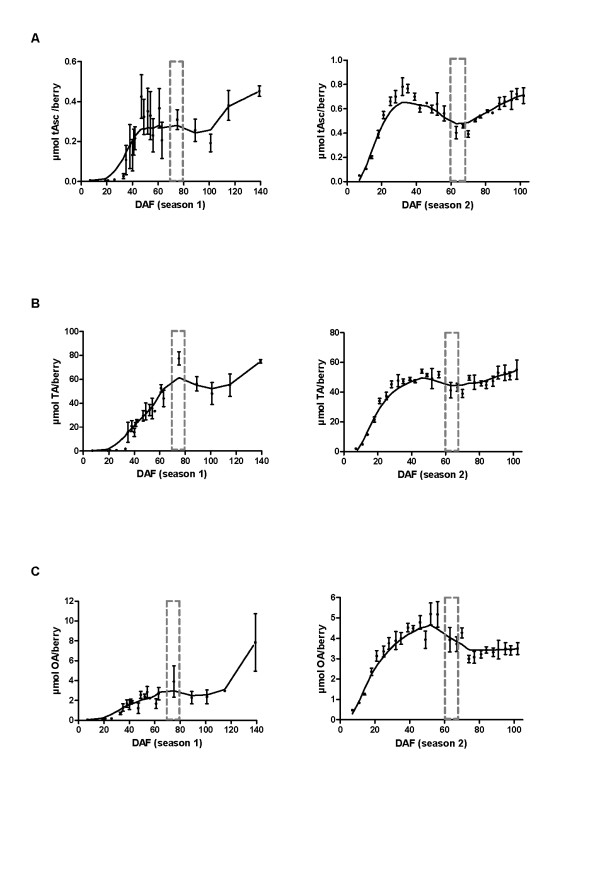
**Accumulation of total ascorbate (tAsc) and the ascorbate catabolites tartaric (TA) and oxalic acids (OA)**. All graphs in the left-hand panel show *Vitis vinifera *c.v. Shiraz berries grown in 2005-2006 (season 1) where *n *= 3 and displaying SEM bars. All graphs in the right-hand panel show *V. vinifera *c.v. Shiraz berries grown in 2007-2008 (season 2) where *n *= 4 and displaying SEM bars. A. Accumulation of tAsc, B. Accumulation of TA, C. Accumulation of OA. The developmental stage of veraison is indicated by a grey dotted box.

Development of season 1 (2005-2006) berries was delayed compared to season 2 (2007-2008) berries. This was evident by the initial delayed increase in fresh berry weight (Additional File 1), a slight delay in the onset of sugar accumulation (Additional File 2) and a 3 week delay in the berry accumulation of maximum levels of malic acid (Additional File 3). Ripening was also delayed in season 1 berries, as the inception of ripening, known as veraison, was approximated at 75 DAF in season 1 and at 60 DAF in season 2. Delayed development may be attributed to the typical seasonal climatic differences such as the cooler maximum and cooler minimum temperatures experienced in mid-November 2006 (season 1) compared to the same period in 2007 (season 2) [60]. A net rate of increase in the accumulation of Asc, TA and OA was evident across c.v. Shiraz berry development (Figure 2). Berries of season 2 accumulated greater maximal quantities of Asc, approximately 1.8 times the content of season 1 berries (Figure 2A). In both seasons, a decrease was evident after the maximum quantity of accumulated Asc was reached. During the latter stages of berry ripening (after 100 DAF in season 1 and after 70 DAF in season 2) a secondary phase of Asc accumulation occurred, restoring the maximum quantity of Asc in the berry by harvest. A comparison of the results of Figures 2A, 2B and 2C clearly demonstrated that berries do not accumulate significant quantities of Asc, particularly when compared to the quantities of accumulated TA and OA, suggesting that compartmental storage of Asc in berries does not occur.

Similarities between the developmental accumulation patterns of Asc and its catabolites, TA and OA were evident. Young berries accumulated TA (Figure 2B), reaching maximum pre-veraison quantities 2 weeks after the attainment of maximum pre-veraison Asc quantities. Berry accumulation of TA was quite stable thereafter in season 2 yet some post-veraison fluctuations were evident in season 1. Berries also accumulated OA in early berry development, however, seasonal differences in the accumulated levels of OA was evident (Figure 2C). The altered sampling strategy of season 2, as detailed in the methods, assisted in minimising the variation of all metabolites investigated in season 1.

### Metabolism of Asc and of Asc biosynthetic precursors

**Figure 3:**
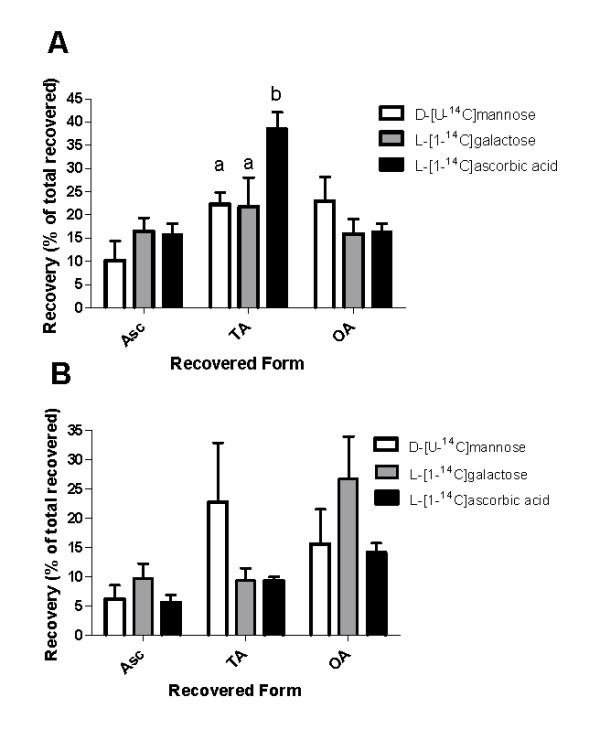
**Recovery of ^14^C-labeled products in grapevine tissue after infiltration of ^14^C-labeled precursors to the excised bunch stem**. Two-way ANOVA with Bonferroni Post-test was performed using GraphPad Prism 5.01 (San Diego, California). The mean values with different letters above the SEM bars indicate significant differences between the proportions of radiolabelled substrates recovered in a specific product (P < 0.05). *V. vinifera *c.v. Shiraz bunches with 3 cm rachis attached were collected at 32 DAF. Data is presented as recovery of each ^14^C-labeled form in either the berry or rachis/stem as a percent of that same ^14^C-labeled form recovered in all tissues. *n *= 4, SEM bars. A. Recovery of ^14^C-labeled products in the berries and B. Recovery of ^14^C-labeled products in the combined rachis and stem tissue.

To identify the existence of a functional Smirnoff-Wheeler Asc biosynthetic pathway in grapevines, the incorporation of radiolabeled carbon from the precursors D-[U-^14^C]mannose, L-[1-^14^C]galactose and L-[1-^14^C]ascorbic acid (L-[1^14^C]Asc) into the products Asc, TA or OA was investigated. Precursors were individually infiltrated into the excised end of a stem, with an intact bunch of grapes attached. After 12 hours of metabolism, labels from D-[U-^14^C]-mannose and L-[1-^14^C]-galactose were incorporated into Asc in both the berries (Figure 3A) and the vegetative (stem/rachis) tissue (Figure 3B). Infiltration of L-[1-^14^C]Asc also resulted in recovery of labeled Asc. Furthermore, metabolism of D-mannose, L-galactose and L-ascorbic acid to form the products TA and OA was demonstrated. Figure 3A shows that L-[1-^14^C]Asc was a more effective precursor of TA in berries than either D-[U-^14^C]mannose or L-[1-^14^C]galactose (P < 0.05) yet in the vegetative tissue each precursor was equally effective for the synthesis of Asc, TA and OA (Figure 3B). However, D-mannose and L-galactose are also involved in other biosynthetic pathways such as the synthesis of structural components, which may influence their availability for incorporation into Asc and downstream metabolites.

### Developmental expression of the Asc biosynthetic pathways and the TA biosynthetic pathway

There were three distinct phases of Asc accumulation in grape berry development observed in both seasons (Figures 2A) but most distinctive in season 2: the pre-veraison (7 to 32 DAF) increase, the pre-veraison (35 to 63 DAF) decrease and the post-veraison (67 DAF to harvest) increase. To investigate whether Asc biosynthetic pathways were developmentally regulated to support these phases of Asc accumulation, and whether this can be correlated to the TA biosynthetic pathway, we conducted quantitative real-time PCR (qRT-PCR) using the berry developmental series of season 2.

Full-length sequences of grapevine genes homologous to those characterised in either the primary or alternative Asc biosynthetic pathway in other plant species were amplified to confirm that the sequences exist in the *V. vinifera *genome. The genes selected for analysis include *GME *encoding GDP-mannose-3,5-epimerase (E.C.5.1.3.18), *Vtc2 *encoding GDP-L-galactose-phosphorylase (*EC unassigned*), *L-GalDH *encoding L-galactose dehydrogenase (*EC unassigned*), *GLDH *encoding L-galactono-1,4-lactone dehydrogenase (EC 1.3.2.3) and *GalUR *encoding D-galacturonic acid reductase (E.C. 1.1.1.203).

**Figure 4:**
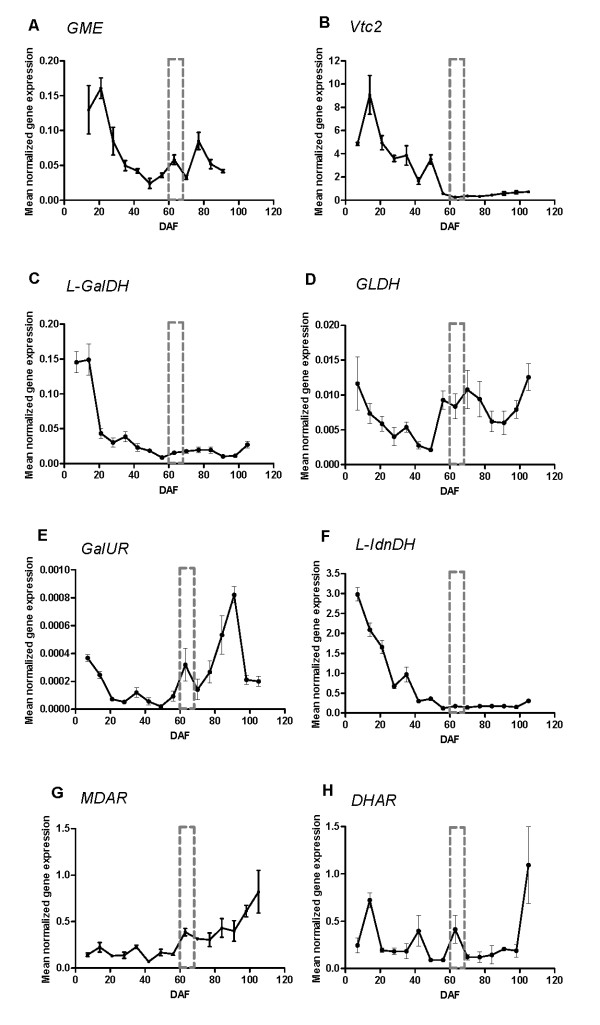
**Transcriptional profiles of selected genes in developing berries, grown in 2007-2008 (season 2)**. Error bars are standard errors of four biological replicates and three technical (qRT-PCR reaction) replicates. Transcriptional changes of *V. vinifera *genes: A. GDP-D-mannose-3,5-epimerase (*GME*), B. GDP-L-galactose phosphorylase (*Vtc2*). C. L-galactose dehydrogenase (*L-GalDH*), D. L-galactono-1,4-lactone dehydrogenase (*GLDH*), E. D-galacturonic acid reductase (*GalUR*), F. L-idonate dehydrogenase (*L-IdnDH*), G. monodehydroascorbate reductase (*MDAR*) and H. dehydroascorbate reductase (*DHAR*). The developmental stage of veraison is indicated by a grey dotted box.

Transcript profiles demonstrated pre-veraison up-regulation of *vvGME *(Figure 4A), *vvVtc2 *(Figure 4B) and *vvL-GalDH *(Figure 4C). However, as the berries ripened, expression of each of these genes was reduced. Specifically from 14 DAF to veraison, the total expression of *vvGME *was down-regulated 3.6-fold, and the expression of *Vtc2 *and *vvL-GalDH *genes were down-regulated at least 16-fold. The expression profile of *vvGLDH*, encoding the enzyme catalysing the final step in Asc biosynthesis, did not correlate with the transcription profiles of the up-stream genes just described; instead the expression of this gene was stable across berry development (Figure 4D). Expression of *vvGalUR *increased with ripening, specifically this gene was up-regulated >2-fold from early development (7 DAF) to ripe stages (91 DAF) (Figure 4E).

The biosynthesis of TA from Asc is known to proceed in grapevines via the activity of L-idonate dehydrogenase (L-IdnDH) [55]. Since our results confirmed TA synthesis from Asc in immature berries (Figure 3A), the total gene expression of *L-IdnDH *was investigated. The results displayed the anticipated pre-veraison up-regulation of this TA biosynthetic gene (Figure 4F).

### The Asc redox state and recycling capacity of developing berries

**Figure 5:**
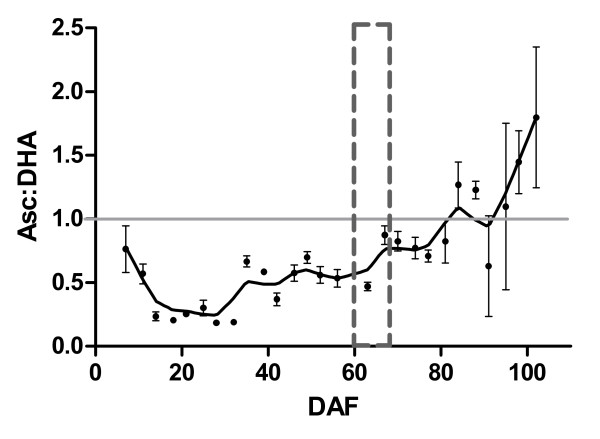
**Accumulation of the redox forms of ascorbate in Shiraz berries across developmental season 2007-2008**. The ratio of reduced ascorbate (Asc) to the oxidised form dehydroascorbate (DHA) is presented, *n *= 4, SEM bars. The graph is fitted with a Lowess curve (medium). The grey horizontal line indicates the developmental stage where the berry tAsc pool is composed of 50% Asc and 50% DHA. The developmental stage of veraison is indicated by a grey dotted box.

Transcription profiles of *vvMDAR *and *vvDHAR *encoding Asc recycling enzymes were investigated in this berry developmental series. Transcription of *MDAR *(Figure 4G) and *DHAR *was up-regulated post-veraison (Figure 4H). There was a >4-fold increase in the expression of *MDAR *and *DHAR *from early development to harvest. Transcription of *DHAR *also increased at specific stages in pre-veraison berries: 14, 42 and 63 DAF. The significant up-regulation of *MDAR *and *DHAR *in post-veraison berries correlates well with the developmental stage where the reduced Asc form contributes greatest to the total ascorbate (tAsc) pool of ripening berries (Figure 5). Although the reduced Asc form predominates in berries at harvest, DHA did contribute to the majority of the tAsc pool for most of development (Figure 5).

## Discussion

Grape berries do not accumulate large quantities of Asc in comparison to other fruits. For example Davey *et al*., [29] reported that blackcurrants (11.2-11.8 μmol/g f.w.), strawberries (3.37 μmol/g f.w.) and kiwifruits (3.41 μmol/g f.w.) are particularly rich in Asc. The results of this current report demonstrated that ripe wine grapes of cultivar Shiraz accumulated Asc (0.43-0.69 μmol/g f.w.) at levels similar to those reported in cranberry (0.67 μmol/g f.w.), apple (0.11-0.56 μmol/g f.w.) and apricots (0.39-0.56 μmol/g f.w.) [29]. It is not known whether low Asc accumulators have a lower rate of Asc biosynthesis, or an increased turnover capacity.

In some species, and at specific physiological stages, Asc catabolism to OA and TA occurs. Oxalate is a common organic acid synthesised in plant tissues to regulate tissue calcium content and to provide protection from herbivory [reviewed in [61]]. Unlike OA, TA does not commonly accumulate in plants. *V. vinifera *berries rapidly synthesise TA during the early cell expansion and growth phase [62], and accumulate TA in the vacuole [63]. Despite this knowledge, the *in-planta *function of TA is still unclear.

The synthesis of OA and TA in plants involves irreversible breakdown of the carbon chain of Asc; however some of the carbon may be recovered in central metabolism. Analysis of TA biosynthesis in Virginia creeper leaves provided evidence that the C2 fragment, possibly as glycoaldehyde, is recycled into products of hexose phosphate metabolism [64]. In OA biosynthesis, L-threonate is recovered from carbons 3 to 6, which is likely to be remetabolised [50,65].

The results of infiltrating the primary Asc biosynthetic pathway intermediates D-[U-^14^C]mannose and L-[1-^14^C]galactose into the excised stem indicate that grapevines have a functional Asc biosynthetic pathway operating *in-planta*. This biochemical evidence was further supported by transcriptional analysis of the grapevine genes homologous to those functioning in the primary Asc biosynthetic pathway in higher plant species. The results of this study demonstrated a positive correlation between the rapid pre-veraison accumulation of Asc in the berries and up-regulation of the Smirnoff-Wheeler Asc biosynthetic genes *vvGME*, *vvL-GalDH *and *vvVtc2*. It is of interest to note the comparatively small changes in transcript levels of some of these major Asc synthetic genes (Figure 4). This suggests the onset of berry TA accumulation is not marked by large-scale synthesis of the respective Asc synthesis enzymes. The subsequent diversion of Asc into a catabolic fate may occur at a generally low rate, but over a sufficient period that TA levels accumulate as seen in the pre-veraison berry, since the TA thus formed is essentially metabolically inert. The correlated expression of *Vtc2 *(referred to as L-galactose-1-phosphate phosphatase) with fruit ripening was also recently demonstrated in tomato [35]. Integration of the biochemical and molecular evidence from this present study indicates that the Smirnoff-Wheeler pathway supports Asc biosynthesis in immature berries.

Despite the developmental evidence of correlative gene expression and Asc accumulation presented here, the mechanisms regulating expression of these Asc biosynthetic genes and activity of the encoded enzymes is yet to be determined in grapevines. Research into the Smirnoff-Wheeler biosynthetic pathway in other higher plants has revealed specific points of regulation. Mieda et al. [66] demonstrated reverse inhibition of spinach L-galactose dehydrogenase by Asc. The concept of feedback regulation at this step in the Asc biosynthetic pathway was also supported by Gatzek et al. [67] who reported that over-expression of the gene encoding L-galactose dehydrogenase in tobacco plants did not result in an increase in leaf Asc content.

Contrary to the developmental regulation of *vvGME*, *vvVtc *and *vvL-GalDH *we demonstrated that *vvGLDH *was not developmentally regulated in berries. Contradictory reports about the correlation of *GLDH *gene expression, its enzyme activity and the Asc content exist. For instance, Tamaoki et al. [33] demonstrated that *GLDH *transcription and *GLDH *activity correlated with the diurnal changes in Asc content of *A. thaliana *leaves. It was also reported that both tAsc content and GLDH activity of potato leaves decreases with aging [68]. However, Bartoli et al. [69] reported that in a range of species there was no clear correlation between Asc content and leaf GLDH protein and activity. In the same report they also demonstrated that wheat leaf Asc content and GLDH activity was relatively constant over the day-night cycle, suggesting that species differences in the diurnal regulation of *GLDH *may exist. The influence of GLDH on Asc was also explored by Alhagdow et al [70] showing that *GalLDH *silencing of *Solanum lycopersicum *plants did not affect the total Asc content but did affect the Asc redox state.

In addition to investigating the primary Asc biosynthetic pathway, we determined a developmental transcription profile of *V. vinifera *D-galacturonic acid reductase, which is homologous to the strawberry NADPH-dependent D-galacturonate reductase gene [20]. Up-regulated expression of *vvGalUR *was demonstrated in post-veraison berries, in agreement with the earlier report of a ripening-associated expression of *GalUR *in strawberry fruit [20]. The post-veraison expression of *GalUR *correlated with a second phase of increased Asc accumulation during berry development, and is suggestive of the existence of a carbon salvage pathway in which Asc is synthesised from a methyl derivative of D-galacturonic acid released during pectin degradation as fruits ripen [29]. Further research into the association between pectin degradation and Asc biosynthesis via this 'salvage' pathway is required. Furthermore, a comparison of the enzymatic rate of GalUR activity with that of the Smirnoff-Wheeler biosynthetic pathway gene-products will provide an insight into the consequences of the comparatively low levels of expression of GalUR as well as the comparatively high levels of *Vtc2 *expression.

There is some evidence to suggest that regulation of the Asc content can occur at the biosynthetic level [reviewed in [71]]. Manipulation of the alternative pathway gene D-galacturonic acid reductase by over-expression in strawberry fruit resulted in a two- to three-fold increase in the total ascorbate content [20]. Attempts to increase the Asc pool size in whole plants via the Smirnoff-Wheeler pathway genes *L-GalDH *and *GLDH *have not been equally successful [67,70]. However, recent studies over-expressing the upstream Smirnoff-Wheeler pathway genes *phosphomannosemutase*, *GME *and *Vtc2 *have resulted in a 2- to 4-fold increase in the foliar Asc content [72-74], which now paves the way for similar transgenic approaches in fruit-bearing plants. In addition, over-expression of the gene encoding the Asc recycling enzyme dehydroascorbate reductase, resulted in a two to four-fold increase in ascorbic acid levels and a significant increase in the redox state of the ascorbate pool in transgenic maize and tobacco [75]. Surprisingly, there have been no studies on the influence of genetic manipulation of *MDAR *despite molecular cloning of plant isoforms [76-79] and purification of a chloroplastic MDAR isoform [80].

Here we describe significant up-regulation of *MDAR *and *DHAR *transcripts in post-veraison berries. The Asc to DHA ratio also increases in berries during this phase of berry development. Increased contribution of the reduced form of Asc to the tAsc pool of berries at the latter stages of ripening could be the result of an increased rate of Asc recycling via the activity of MDAR and DHAR and/or an up-regulation of the alternative 'salvage' pathway. The high DHA content in immature berries of this study may support TA formation in the early physiological stages of development; indeed we have shown a timely up-regulated total expression of the TA biosynthetic gene *L-IdnDH*. Developmental expression pattern of *L-IdnDH *reported here supports that originally reported by DeBolt et al. [55]. The more frequent time-point analysis of *L-IdnDH *transcription presented here enabled us to determine that *L-IdnDH *was up-regulated from 7 DAF. This transcription profile of *L-IdnDH *indicates that TA biosynthesis may occur as early as bud-break. Hancock et al. [46] demonstrated that blackcurrant (*Ribus nigrum L*.) flowers have the capacity to synthesise Asc; the potential for Asc biosynthesis and degradation to TA in floral organs of grapevines must therefore be explored.

In this report we have demonstrated that in immature berries turnover of L-[1-^14^C]Asc to TA and OA and recycling of Asc is evident after 12 hours of metabolism. Franceschi and Tarlyn [41] demonstrated that 75 to 80% of the label of L-[1-^14^C]Asc could be recovered in the form of Asc after 12 hours in *Arabidopsis *and *Medicago*. Their results suggest that whilst some turnover of Asc is apparent, the majority of Asc is recycled. In grapevines, however, the turnover is more rapid than the recycling of Asc, visualised by the recovery of more than twice the proportion of ^14^C label from L-[^14^C]Asc in the catabolic forms of TA and OA compared to that in Asc. Research into the involvement of Asc in multiple parallel metabolic pathways is some-what limited by the current ^14^C radiotracer techniques available. ^13^C metabolic flux analysis may prove to be a more effective tool for quantification of the flux of complex metabolic pathways [81], and should in the near-future be employed to the study of Asc metabolism in fruit.

In previous research we have shown that leaves accumulate higher quantities of Asc and have a higher Asc to DHA ratio than berries at any stage of maturation investigated [59]. Translocation of these ample Asc pools to support TA and OA accumulation in berries is presently unsubstantiated. It is however well established that grape berries accumulate assimilates translocated from the leaves during post-veraison development; for example sucrose produced by photosynthesis in the leaf is translocated to the berry via the phloem [82]. Translocation of Asc from leaves to fruits or tubers via the phloem has been demonstrated in other plant species [32,42,43]. However, the total Asc accumulation in blackcurrant fruits was shown to be the result of a high biosynthetic capacity and low rate of Asc turnover rather than import via the phloem [46]. It therefore remains to be determined if foliar Asc contributes to the accumulation of Asc in grape berries, and if the secondary rate of Asc accumulation observed in post-veraison berries is an indicator of long-distance Asc translocation.

## Conclusion

Here we report developmental regulation of the biosynthetic genes *vvGME*, *vvVtc2 *and *vvL-GalDH*, the recycling genes *vvDHAR *and *vvMDAR *and of the catabolic gene (or TA biosynthetic gene) *L-IdnDH *in berries. The results demonstrated that immature berries have up-regulated expression of Asc biosynthetic genes, a rapid rate of Asc accumulation, and are capable of *in-situ *Asc biosynthesis via the primary Smirnoff-Wheeler Asc biosynthetic pathway. The generally low level of change in transcript abundance seen during berry development may be explained by proposing that the diversion of L-Asc metabolism to support TA synthesis is small, and that the 'terminal' nature of TA as a metabolite leads to its gradual accumulation. Further radiotracer studies may in the future provide the quantitative metabolite data to back-up this molecular work. In contrast to this early diversion of Asc metabolism, ripe berries were shown to have up-regulated expression of the recycling genes, and of the alternative 'salvage' pathway gene *GalUR*, which correlated with both the secondary rate of Asc accumulation and an increased contribution of reduced Asc to the total Asc pool. Turn-over of L-[1-^14^C]Asc to TA in immature berries was observed, with some Asc recycling. We propose that the flux of Asc during early berry development is diverted towards the synthesis of TA and OA, and thereafter returns to non-synthetic, redox-associated roles.

## Methods

### Plant material and growth conditions

*Vitis vinifera *cultivars Shiraz clone BVRC12 on Shwarzmann rootstock were grown at the University of Adelaide Coombe vineyard in the Adelaide plains (South Australia) at 123 m elevation and latitude of 34°58'S. These vines were planted in 1993 with 3 m row spacing and 1.8 m vine-spacing. The vines were spur-pruned by hand to between 30 and 40 nodes per vine. These vines were used for all experiments. All plant material used in this study was immediately snap-frozen on site in liquid nitrogen and stored at -80°C for analysis.

### Sampling Regime

In the 2005-2006 developmental season (season 1) bunches from three replicate vines were randomly sampled during development. However, some variability was observed between the physiological development of bunches. Therefore, the selection regime was improved in the 2007-2008 season (season 2) by sampling from bunches at the same physiological stage of development. This was achieved by tagging individual bunches across all vines at 50% cap-fall. In season 2, bunches from four vine replicates were tagged. These four vine replicates were repeated across five rows, i.e. sampling of replicate 1 was the pooled berries from five vines, each randomly positioned across five separate rows. Since ripening berries represent a major carbohydrate sink, minimising the number of berries removed from a bunch reduces variability of the sink-strength of the bunch.

The first sampling point in season 1 was 7 days after flowering (DAF) and then once the berries were large enough, sampling was conducted 3 times per week. After veraison, sampling was reduced to once per week due to an observed reduction in the accumulation of the metabolites of interest. In season 2, grape berries were sampled twice per week throughout the season. The sampling season was shortened from 139 DAF in season 1 to 105 DAF in season 2 due to the accelerated rate of development and ripening of season 2 berries.

### Berry developmental parameters

Sampled berries (10 berries at the pre-veraison and 50 at the post-veraison time-points) were thawed at room temperature and blot dried to remove excess liquid before weighing. These berries were subsequently crushed and an aliquot of the clear juice was used to determine total soluble solids (TSS) with a pre-calibrated refractometer.

### Metabolite extractions and analyses

Asc, DHA, TA, OA and MA were extracted and analysed by RP-HPLC as described in [59].

### Identification of gene sequences

Full length sequences were obtained from the National Centre for Biotechnology Information (NCBI) database. In the absence of full length sequences, expressed sequence tags (ESTs) and tentative consensus (TC) sequences were mined from either the NCBI database or The Institute for Genomic Research (TIGR) Grape Gene Index database. The forward primer used to amplify L-galactono-1,4-lactone dehydrogenase was designed from the genomic sequence AM443025. All other primers were designed based on the fragments or full-length mRNA or cDNA sequences available in the databases. The complete coding sequences of the *V. vinifera *dehydroascorbate reductase (EU280162), D-galacturonic acid reductase (DQ843600), L-idonate dehydrogenase (DQ124868) and GDP-L-galactose-phosphorylase (AM485812) were available from the NCBI database; the full coding sequences were therefore not amplified in this present study. Genes were amplified using Platinum *Taq *DNA polymerase high-fidelity (Invitrogen, Victoria, Australia) according to the conditions listed in Additional File 4. RNA derived from young, green berries (c.v Shiraz) was used for cDNA synthesis of each of the genes except for monodehydroascorbate reductase and D-galacturonic acid reductase where RNA derived from ripe berries was used as the template. Berry-derived RNA was reverse transcribed using the SuperScript III First-Strand cDNA Synthesis Kit (Invitrogen) with the oligo (dT)_20 _primer according to the manufacturer's instructions. PCR products were gel purified using the Wizard PCR Preps DNA Purification System (Promega, NSW, Australia) and cloned into pTOPO2.1 PCR cloning vector (Invitrogen) according to manufactures' instructions. Gene sequences were confirmed by sequencing the cloned products with M13F and M13R primers (Invitrogen).

### RNA extraction and cDNA synthesis

Total RNA was extracted from grape berries and leaves using the sodium-perchlorate method as described by Rezaian and Krake [83] with modifications by Davies and Robinson [84]. Total RNA was further purified and DNase treated using an RNeasy Mini Kit (Qiagen, Victoria, Australia) and an RNase-Free DNase Set (Qiagen) according to the manufacturer's instructions. The quality of the DNase-treated RNA (1 μg) was determined by visualising intact ribosomal bands with agarose gel electrophoresis after treatment of the sample with deionised formamide, and by the absorbance ratio of 280 nm to 260 nm of ≥ 2. RNA samples with absorbance ratio of 260 nm to 230 nm < 2 (indicating polysaccharide contamination) were precipitated and concentrated as described by Davies and Robinson [84]. Berry-derived RNA (1.5 μg) was reverse transcribed using the SuperScript III First-Strand cDNA Synthesis Kit (Invitrogen) with the oligo (dT)_20 _primer and according to the manufacturer's instructions. cDNA reactions were diluted 10-fold to the final volume of 200 μl with 10 mM Tris-HCl, pH 7.6.

### Quantitative real-time PCR (qRT-PCR) analysis of gene transcription

Quantitative analysis of gene transcription was determined by qRT-PCR using the SYBR green method on the iCycler (Bio-Rad Laboratories, Life Science, NSW, Australia). The thermal cycling conditions were Cycle 1 (95°C for 2 min), 35 cycles of Cycle 2 (95°C for 30 sec, 57°C for 30 s and 72°C for 15 s), Cycle 3 (95°C for 30 s), followed by a melt cycle of 0.5°C increments per 30 s from 57°C to 95°C. The SYBR green Supermix (BioRad Laboratories) was used as per the manufacturer's instructions; each reaction contained 1× Supermix, 0.2 μM primer and 3 μl of the diluted cDNA, in a total volume of 20 μl. Each reaction was performed in triplicate. The melt-curve analysis was conducted to confirm amplicon purity. The primer pairs designed (Additional File 5) amplified single copy genes in all cases except those designed to amplify both homologs of L-idonate dehydrogenase and both homologs of GDP-D-mannose-3,5-epimerase. Before conducting qRT-PCR, each PCR product was visualised by gel electrophoresis and sequences confirmed by sequencing the cloned product. The suitability of both Ubiquitin (BN00705) and Elongation Factor 1α (EC 959059) as gene references were tested on cDNA from the berry developmental series (data not shown). Elongation factor 1α was selected due to its more stable expression across both these series. Each reaction was performed in triplicate. BioRad iQ 3.0 Real Time PCR detection system software was used to observe the melt curve profiles, and to measure the primer pair amplification efficiencies. Q-Gene software [85] was used to calculate the mean normalized gene expression of each gene against each cDNA tested relative to the reference gene, see Equation 3 in table two of Muller et al. [85].

### Radiotracer experiments

Grape bunches of approximate weight of 15 g with at least 3 cm of stem were excised from the vine under water in order to avoid cavitation of the phloem. These bunches were collected at the pre-veraison physiological stage (32 DAF) when Asc is rapidly accumulating. The excised end of the stem was briefly blot dried before transfer into a 100 μl tube containing either one of the three precursor treatments. The treatments were 1 μCi^14^C-D-mannose (American Radiolabeled Chemicals, St Louis, MO), 1 μCi ^14^C-L-galactose (American Radiolabeled Chemicals, St Louis, MO) and 0.5 μCi ^14^C-L-ascorbic acid (GE Healthcare-Amersham Radiolabels, U.K.). Each treatment was prepared in 20 mM MES pH 5.0. Four replicates were used for each treatment. An additional 100 μl of 20 mM MES pH 5.0 was added to each tube after 1.5 and 3 hours of metabolism. After 12 hours of metabolism, bunches were removed from the tubes and 2 cm of the infiltrated stem was excised and discarded. The berries were collected, weighed and snap-frozen. The rachis and 1 cm of the remaining stem were weighed and snap-frozen together. Asc, TA and OA were extracted and analysed from 1 g of tissue using the same methods as for the extraction of unlabelled metabolites described in [59] with all changes and additions described here. A 2 ml aliquot of the extract was concentrated to 1 ml in a rotary evaporator (LABCONCO Centrivap concentrator, Missouri, USA) under reduced pressure at 35°C for 18 hours. The metabolites were separated by HPLC using a System Gold HPLC with the software 32 Karat (Beckman Coulter, NSW, Australia). Aliquots of the post-column eluate were collected into 5 ml scintillation vials according to the peaks observed on a flatbed recorder (Kipp and Zonen, Delft, The Netherlands) directly connected to the photodiode array detector. The successful collection of individual products according to visualization from the chart recorder was confirmed by injection of a mixed non-radioactively-labeled standard of 20 μM Asc, 0.15 mM TA and 0.11 mM OA, and collecting the post-column eluate for re-analysis and visualization of a single peak on the chromatogram output (data not shown). The radioactivity of each grapevine extract eluate was determined using a Canberra Packard TriCarb 2100TR (Canberra-Packard, USA) liquid scintillation counter, each sample was counted over 5 min with the average recorded.

## Abbreviations

**Asc**: ascorbate; **c.v**.: cultivar; **DAF**: days after flowering; **DHA**: dehydroascorbate; **DHAR**: dehydroascorbate reductase; **EDTA**: ethylenediaminetetraacetic acid; **FW**: fresh weight; **GLDH**: L-galactono-1,4-lactone dehydrogenase; **GalUR**: D-galacturonic acid reductase; **GME**: GDP-mannose-3,5-epimerase; **HPLC**: high performance liquid chromatography; **L-GalDH**: L-galactose dehydrogenase; **L-IdnDH**, L-idonate dehydrogenase; **MA**: malic acid; **MDAR**: monodehydroascorbate reductase; **OA**: oxalic acid; **qRT-PCR**: quantitative real-time polymerase chain reaction; **TA**: tartaric acid; **tAsc**: total ascorbate; **Vtc2**: GDP-L-galactose-phosphorylase; ***vv***: *Vitis Vinifera L*.

## Authors' contributions

VJM designed and conducted all research experiments, analysed the data, and drafted/constructed the manuscript. CMF supervised all research. CMF and KLS contributed to the research ideas and design, and the editing of the manuscript.

## Supplementary Material

Additional file 1 **The mean fresh weight of berries across development**. A. 2005-2006 developmental season, *n *= 3, SEM bars and B. 2007-2008 developmental season, *n *= 4, SEM bars. Lowess curves were fitted to both graphs A and B. The developmental stage of veraison is indicated by a grey dotted box. Click here for file

Additional file 2 **Total Soluble Solids (TSS) expressed as Brix° of berries across development**. A. 2005-2006 developmental season, *n *= 3 and SEM bars. B. 2007-2008 developmental season, *n *= 4, SEM bars, a lowess curve was fitted to the graph. The developmental stage of veraison is indicated by a grey dotted box. Click here for file

Additional file 3 **Malic acid accumulation in developing berries]**. A. 2005-2006 developmental season. n = 3, SEM bars and B. 2007-2008 developmental season *n *= 4, SEM bars. Lowess curves were fitted to both graphs A and B. The developmental stage of veraison is indicated by a grey dotted box. Click here for file

Additional file 4 **List of Primers used in amplification of full-length coding sequences**. The table lists primer sequences, GenBank accession numbers and PCR conditions used in the amplification of genes using template cDNA derived from RNA of *Vitis vinifera *c.v. Shiraz berries. Click here for file

Additional file 5 **List of Primers used in quantitative real-time PCR (qRT-PCR) reactions**. The table lists the primer sequences used in qRT-PCR reactions and the length of the amplicon in base pairs (bp). These reactions enabled analysis of gene transcription against berry cDNA template. Click here for file
