# Supplementary figures and images for: Ascorbate metabolism and the developmental demand for tartaric and oxalic acids in ripening grape berries
Source: BMC Plant Biol. 2009 Dec 9;9:145. doi: 10.1186/1471-2229-9-145 (PMC2797797; doi:10.1186/1471-2229-9-145)

**A**

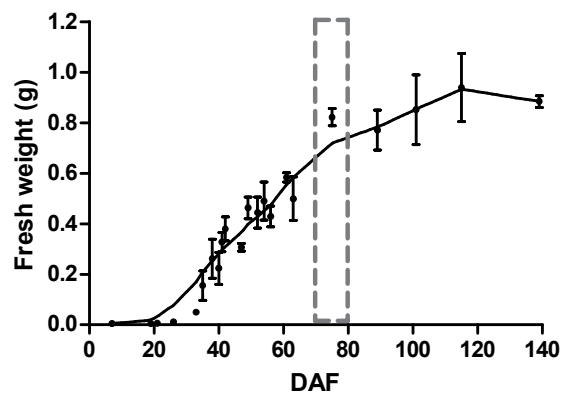

**B**

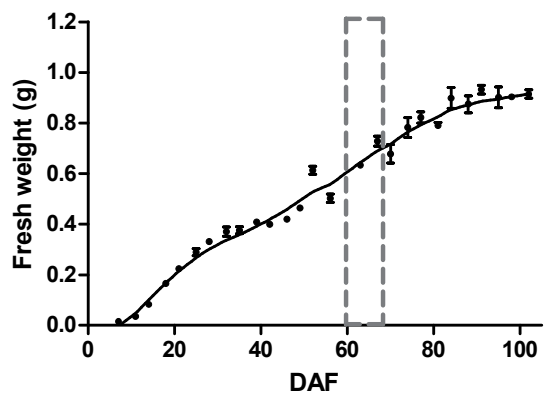

Supplement: Additional file 1 — The mean fresh weight of berries across development. A. 2005-2006 developmental season, n = 3, SEM bars and B. 2007-2008 developmental season, n = 4, SEM bars. Lowess curves were fitted to both graphs A and B. The developmental stage of veraison is indicated by a grey dotted box. [file 1471-2229-9-145-S1.PDF]

A

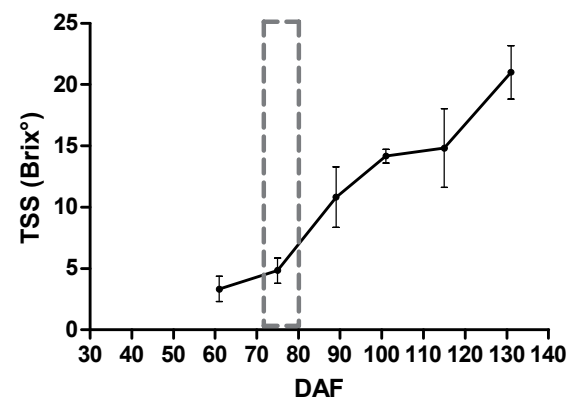

B

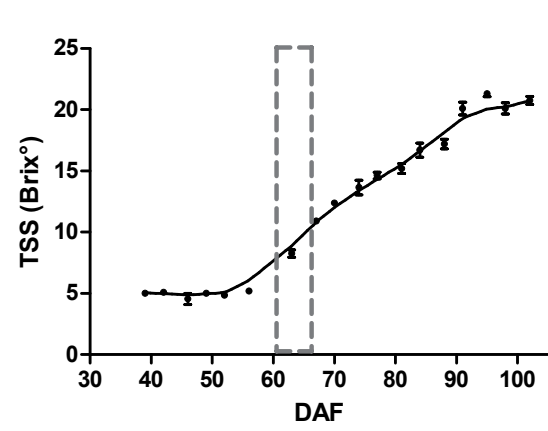

Supplement: Additional file 2 — Total Soluble Solids (TSS) expressed as Brix° of berries across development. A. 2005-2006 developmental season, n = 3 and SEM bars. B. 2007-2008 developmental season, n = 4, SEM bars, a lowess curve was fitted to the graph. The developmental stage of veraison is indicated by a grey dotted box. [file 1471-2229-9-145-S2.PDF]

**A**

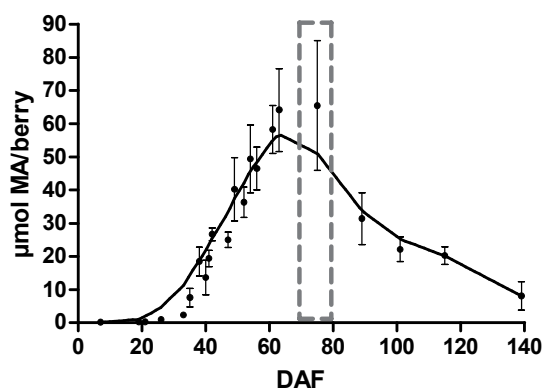

**B**

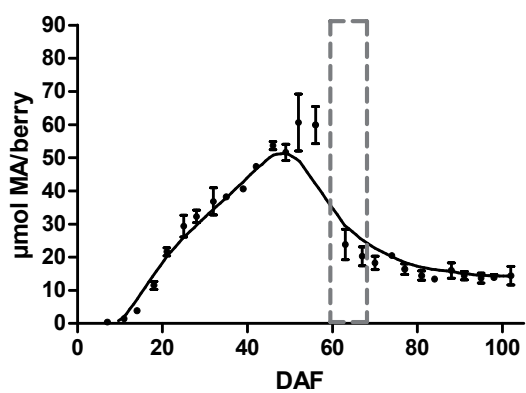

Supplement: Additional file 3 — Malic acid accumulation in developing berries]. A. 2005-2006 developmental season. n = 3, SEM bars and B. 2007-2008 developmental season n = 4, SEM bars. Lowess curves were fitted to both graphs A and B. The developmental stage of veraison is indicated by a grey dotted box. [file 1471-2229-9-145-S3.PDF]
