# Supplementary material for: Ascorbate metabolism and the developmental demand for tartaric and oxalic acids in ripening grape berries
Source: BMC Plant Biol. 2009 Dec 9;9:145. doi: 10.1186/1471-2229-9-145 (PMC2797797; doi:10.1186/1471-2229-9-145)
Supplement: Additional file 4 — List of Primers used in amplification of full-length coding sequences. The table lists primer sequences, GenBank accession numbers and PCR conditions used in the amplification of genes using template cDNA derived from RNA of Vitis vinifera c.v. Shiraz berries. [file 1471-2229-9-145-S4.PDF]

Gene	Accession Number	Sequence of Reverse (R) and Forward (F) Primers	Annealing Temperature (˚C)/ Mg concentration (mM)
Monodehydroascorbate reductase	EF554360	**F** 5’TGCAGATACATTCATCGATCGG3’	55/2
		**R** 3’GATCACATAACAGCCCTGAAGG5’	
L-galactono-1,4-lactone dehydrogenase	FJ772444	**F** 5’CTACTTCGCTCTTCTCCTTGG3’	57/2
		**R** 3’GAAGCTTCTCAAATGGTATCCG5’	
GDP-D-mannose-3,5-epimerase	EF554358	**F** 5’CCCTTGAGAGCTTCACATTATC3’	55/2
		**R** 3’ATGGCATAAGCTACCAGAGC5’	
L-galactose dehydrogenase	EF554359	**F** 5’TGCAGGAGCACACCTTCCTTC3’	50/2.5
		**R** 3’GGACCAGGAAAGTCGAGCAG5’	
