# Supplementary material for: Ascorbate metabolism and the developmental demand for tartaric and oxalic acids in ripening grape berries
Source: BMC Plant Biol. 2009 Dec 9;9:145. doi: 10.1186/1471-2229-9-145 (PMC2797797; doi:10.1186/1471-2229-9-145)
Supplement: Additional file 5 — List of Primers used in quantitative real-time PCR (qRT-PCR) reactions. The table lists the primer sequences used in qRT-PCR reactions and the length of the amplicon in base pairs (bp). These reactions enabled analysis of gene transcription against berry cDNA template. [file 1471-2229-9-145-S5.PDF]

Gene	Sequences of Forward (F) and Reverse (R) Primers	Amplicon (bp)
L-galactose dehydrogenase	**F** 5’CAACTTTTTCGACACCTCTCC3’	139
	**R** 3’ATCAAAGCCCTCAGCATACC5’	
GDP-D-mannose-epimerase	**F** 5’GTGCTGGTGGTTTTATTGC3’	94
	**R**3’ATGTGCTCGTTCTTCTTCC5’	
GDP-L-galactose-phosphorylase	**F** 5’ATATTTCCGGTTGGGTTACA3’	126
	**R**3’TGCAGTGGTTATTTTCCTAGTT5’	
L-galactono-1,4-lactone dehydrogenase	**F** 5’GCCACCTACTACTCCTTCCC3’	122
	**R** 3’ACTTCATGGGTTCCACTCC5’	
Elongation Factor 1α	**F** 5’GCAAGGAGCTTGAGAAAGAG3’	129
	**R** 3’GAACAGCAAAACGACCAAG5’	
Ubiquitin	**F** 5’GCAGGAAAAAGAAGTGTGGC3’	115
	**R** 3’AATGCTCCCCGTGTGTAAC5’	
Monodehydroascorbate reductase	**F** 5’GACAGGTGGAAGAGGAGAAA3’	101
	**R** 3’CAGTGGGAAAGTAGCAACATC5’	
L-idonate dehydrogenase	**F** 5’AAGTTTGCCTTGTGGGTTTG3’	205
	**R** 3’AAGGCTTCCTCCACATCCTT5’	
Dehydroascorbate reductase	**F** 5’CCTACAAGATGCATCTGATCAA3’	127
	**R** 3’CCAGTAATGACATCAGAGTCAG5’	
D-galacturonic acid reductase	**F** 5’TGATGTCCTGGAAGATATCGC3’	128
	**R** 3’GGTTCTGTTTCATTCTCTCCTTG5’	
